# NLRP3 inflammasome in major swine viral infections: Host defense and viral antagonism

**DOI:** 10.1080/21505594.2026.2753323

**Published:** 2026-10-29

**Authors:** Ya-Di Guo, Hang-Tian Mei, Yang Wang, Yu-Xin Xiang, Ying-Nan Li, Guo-Yu Yang, Jiang Wang, Lei Zeng, Bei-Bei Chu, Sheng-Li Ming

**Affiliations:** a College of Veterinary Medicine, Henan Agricultural University, Zhengzhou, Henan Province, China; b Key Laboratory of Animal Biochemistry and Nutrition, Ministry of Agriculture and Rural Affairs, Zhengzhou, Henan Province, China; c Key Laboratory of Veterinary Biotechnology of Henan Province, Henan Agricultural University, Zhengzhou, Henan Province, China; d Ministry of Education Key Laboratory for Animal Pathogens and Biosafety, Henan Agricultural University, Zhengzhou, Henan Province, China; e Longhu Advanced Immunization Laboratory, Henan Agricultural University, Zhengzhou, Henan Province, China; f International Joint Research Center of National Animal Immunology, Henan Agricultural University, Zhengzhou, Henan Province, China

**Keywords:** NLRP3 inflammasome, pyroptosis, swine viral infections, viral immune evasion, ubiquitin–autophagy axis

## Abstract

The NLRP3 inflammasome is a cytosolic signaling platform that drives caspase-1-dependent maturation of IL-1β and IL-18 and gasdermin D (GSDMD)-mediated pyroptosis. During infection with major porcine viruses – including African swine fever virus, porcine reproductive and respiratory syndrome virus, pseudorabies virus, porcine epidemic diarrhea virus, transmissible gastroenteritis virus, and porcine circovirus type 2, NLRP3 exerts opposing effects: moderate activation restricts viral propagation, whereas sustained stimulation drives tissue injury. We review how structurally unrelated viral proteins converge on limited upstream events – endolysosomal damage, ion flux, mitochondrial injury, ER stress, and the ubiquitin–autophagy network. Because genetically distinct porcine pathogens exploit this same regulatory axis, it represents a shared point of vulnerability. We assess therapeutic options targeting NLRP3 assembly, GSDMD pore formation, and host proteostasis, and highlight questions requiring validation in porcine cells and pigs.

## 1. Introduction

Pigs serve as both reservoir and amplification hosts several viral pathogens of major economic importance, including ASFV, PRRSV, PRV, PEDV, TGEV, and PCV2, which together cause severe losses in global swine production [1–6]. Innate immunity constitutes the first line of host defense, relying on pattern recognition receptors (PRRs) including Toll-like receptors (TLRs), RIG-I-like receptors (RLRs), and NOD-like receptors (NLRs) to detect pathogen- and damage-associated molecular patterns (PAMPs and DAMPs) [7–10]. Once these cues are recognized, PRRs trigger signaling cascades that often converge on inflammasome activation.

Inflammasomes are cytosolic multiprotein complexes that link danger sensing to inflammatory output and host defense. Structurally, they typically consist of a sensor protein, the adaptor molecule ASC, and the effector procaspase-1. Among these, the canonical NLRP3 inflammasome comprises the sensor NLRP3, the adaptor ASC, and the effector procaspase-1 [11,12]. Following assembly, active caspase-1 cleaves pro-IL-1β and pro-IL-18 into their mature secreted forms and processes GSDMD to generate an N-terminal fragment that oligomerizes into plasma membrane pores, executing pyroptosis, a lytic and pro-inflammatory form of cell death [13,14].

NLRP3 differs from the other inflammasome sensors and is the most extensively characterized of them, largely because it responds to a wide range of cellular stress cues. Unlike NLRP1, which primarily responds to dsRNA and undergoes functional degradation, or AIM2, which directly recognizes cytosolic dsDNA via its HIN domain, NLRP3 does not recognize a defined ligand but instead integrates generic stress signals, including potassium efflux, calcium flux, lysosomal rupture, mitochondrial dysfunction, and reactive oxygen species (ROS) production [15–19]. This breadth allows NLRP3 to support host defense against many metabolic and infectious perturbations, and it also leaves the sensor open to manipulation by pathogens.

NLRP3 activation is controlled by a two-signal cascade that prevents spontaneous inflammation. Priming (Signal 1), typically mediated by NF-κB, upregulates NLRP3 and pro-inflammatory cytokines, licensing the cell for subsequent activation [20]. Subsequently, activation (Signal 2), triggered by ATP, ionic disturbances, or organelle damage, promotes NLRP3 oligomerization, ASC speck formation, and caspase-1 activation [21]. Signal 2 has no dedicated receptor; instead it integrates stress signals from several organelles, including lysosomal membrane permeabilization leading to cathepsin release [22,23], mitochondrial dysfunction characterized by membrane potential loss and mtDNA efflux [24,25], metabolic reprogramming affecting glycolytic enzymes [26,27], and ion-flux disruption, in particular K^+^ efflux, which licenses NEK7 binding to NLRP3 [28]. Non-canonical pathways use different effector caspases: caspase-4/5/11 cleave GSDMD and can secondarily engage the NLRP3 axis [29–31]. This route is well defined in Gram-negative bacterial infection through cytosolic LPS sensing, but its contribution to viral infection has not been analyzed systematically. Viral disruption of endosomal escape or membrane integrity may mimic the membrane perturbation caused by bacterial LPS and thereby engage the non-canonical axis.

NLRP3 sits at the center of host–pathogen interactions during swine viral infection. DNA viruses (ASFV [32,33], PRV [34]) encode proteins that directly interface with inflammasome machinery, whereas RNA viruses (PRRSV [35,36], PEDV [37,38], TGEV [39]) largely modulate NLRP3 indirectly through cellular stress pathways – ER stress, mitochondrial damage, and ionic imbalance. PCV2, despite its minimal genome, reprograms host microRNA networks to amplify inflammasome signaling [40]. Some pathogens dampen NLRP3 activity to avoid immunosurveillance, whereas others hyperactivate it and cause tissue damage. Mechanistic insight has advanced quickly, but most of it comes from non-porcine systems, and swine-specific validation is still incomplete. This review brings together current findings on the major porcine viruses, the viral strategies that target inflammasome activation, and the translational implications for veterinary and human medicine.

## 2. NLRP3 inflammasome: structure, activation, and regulation

### 2.1. Architecture, assembly, and canonical vs non-canonical activation

NLRP3 contains an N-terminal pyrin domain (PYD), a central NACHT domain with ATPase activity, and a C-terminal leucine-rich repeat (LRR) domain [11]. In resting cells, intramolecular interactions maintain an autoinhibited state. Priming signals, transduced largely via NF-κB, elevate NLRP3 expression and license post-translational modifications required for activation [20,41,42]. Upon stimulation, NLRP3 undergoes NACHT-dependent oligomerization and recruits ASC via homotypic PYD–PYD interactions. ASC polymerizes into filamentous structures that cluster procaspase-1 through CARD–CARD interactions, driving proximity-induced activation [43]. Structural studies have shown how small-molecule inhibitors stabilize the inactive NACHT conformation, which provides a basis for therapeutic targeting [44] (Figure 1).

**Figure 1.**
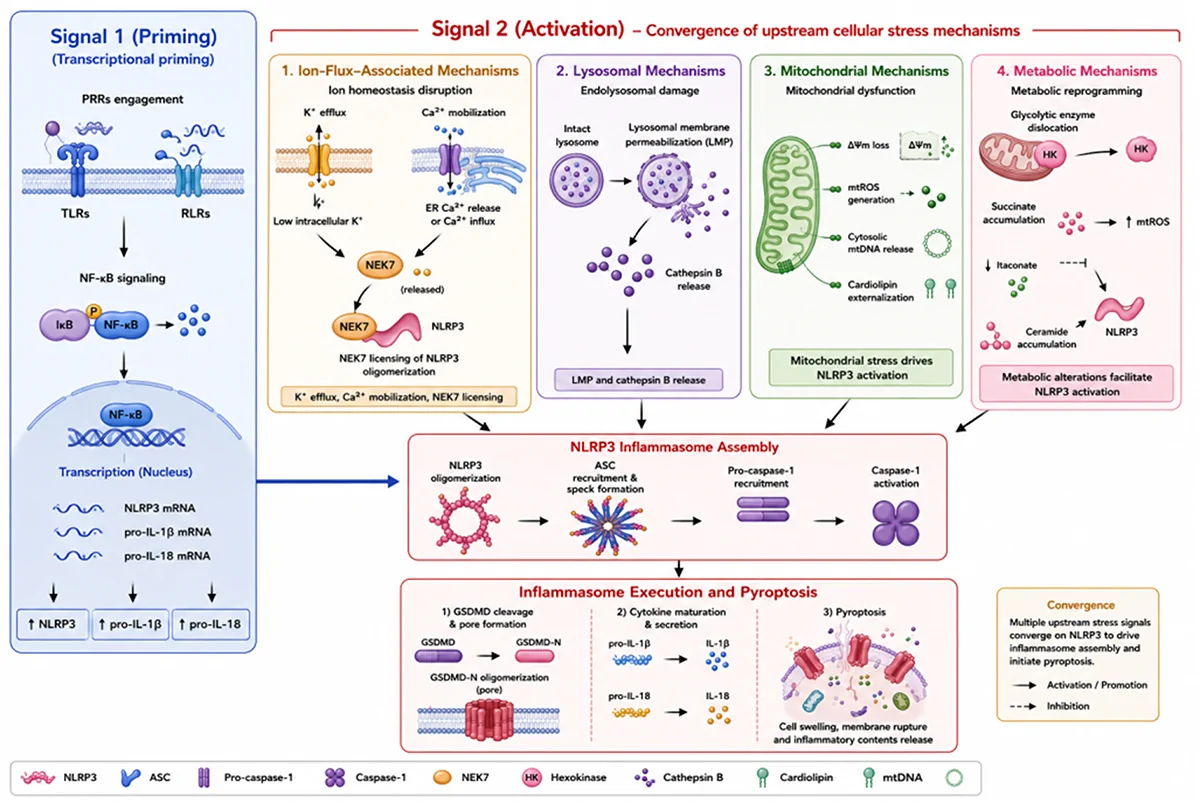
Upstream signaling pathways of NLRP3 activation. NLRP3 inflammasome activation is tightly regulated by a two-signal model. Signal 1 (priming, blue, left) involves pattern recognition receptor engagement, leading to NF-κB-dependent transcription of NLRP3 and pro-inflammatory cytokines. Signal 2 (activation) is triggered by multiple upstream cellular stress mechanisms: (1) Ion flux (top left): Disruption of ionic homeostasis, particularly K^+^efflux and Ca^2+^mobilization, liberates NEK7 to promote NLRP3 oligomerization; (2) Lysosomal disruption (top right): Endolysosomal damage and lysosomal membrane permeabilization (LMP) release cathepsin B into the cytosol; (3) Mitochondrial dysfunction (bottom right): Loss of membrane potential (ΔΨm), generation of mtROS, and release of mtDNA into the cytosol; (4) Metabolic stress (bottom left): Metabolic reprogramming, including glycolytic enzyme (e.g. hexokinase) dislocation and accumulation of metabolic intermediates (e.g. succinate, ceramide), further facilitates activation. These signals drive NLRP3 oligomerization, ASC speck assembly, and caspase-1 activation, resulting in GSDMD-mediated pyroptosis and cytokine maturation.

Canonical NLRP3 activation during viral infection integrates multiple upstream signals. Potassium efflux, often mediated by viral viroporins or membrane disruption, liberates NEK7 to license NLRP3 oligomerization [18,28,45,46]. Concurrently, mitochondrial dysfunction generates mtROS that oxidatively modify NLRP3 to promote activation [15,24,47,48]. Lysosomal disruption following viral particle uptake releases cathepsin B, further contributing to inflammasome assembly [49]. These signals often act synergistically. For instance, cytosolic mitochondrial DNA (mtDNA), released from damaged mitochondria, functions as a DAMP that directly facilitates NLRP3 assembly [47]. This mechanism is prominent in PEDV infection and likely represents a common route by which RNA viruses engaging mitochondrial stress activate NLRP3 (Figure 1).

Non-canonical inflammasome pathways intersect with NLRP3 signaling. In mammals, caspase-11 (or caspase-4/5 in humans) senses cytosolic lipopolysaccharide, triggering GSDMD cleavage and K^+^ efflux that secondarily activates NLRP3 [50–53]. Although swine viral pathogens lack LPS, viral disruption of endosomal membranes or concurrent bacterial co-infections in the respiratory and enteric tracts may permit cytosolic LPS translocation or direct caspase-11/4/5 engagement. Whether ASFV or PRRSV directly activate the non-canonical caspase pathway in porcine macrophages has not been tested, and this question should be addressed (Supplementary Table 1).

### 2.2. Endolysosomal damage, ubiquitination, and autophagy as upstream checkpoints

Endolysosomes are not only degradative compartments; they also serve as platforms that couple pathogen sensing to inflammasome activation [54]. Endolysosomal damage, cathepsin release, ion fluxes, and endolysosomal trafficking are well-established upstream events contributing to NLRP3 activation [55]. Viral entry through endocytosis often perturbs endosomal integrity, leading to lysosomal membrane permeabilization and the release of cathepsin B into the cytosol, which directly facilitates inflammasome assembly [56–58]. Intracellular compartmentalization therefore determines how efficiently endosomal escape feeds into the cytosolic NLRP3 platform.

Ubiquitination provides critical post-translational control of NLRP3. K63-linked ubiquitination (e.g. by TRAF3) promotes inflammasome assembly, whereas K48-linked ubiquitination (e.g. by MARCH7, FBXL2) targets NLRP3 for proteasomal degradation [59,60]. The deubiquitinase BRCC3 removes activating ubiquitin modifications, modulating activation thresholds [41]. Porcine viruses manipulate these enzymes to either potentiate or restrain inflammasome output.

Autophagy regulates NLRP3 at multiple levels. Mitophagy eliminates damaged mitochondria, reducing mtROS and limiting activation [61–63]. Conversely, autophagy contributes to the resolution of inflammation by mediating secretion of IL-1β and the clearance of inflammasome components, including ASC specks [63–65]. Disrupting autophagic flux prolongs inflammasome activity. Multiple swine viruses modulate mitophagy and autophagy to calibrate NLRP3 responses during infection (Figure 1).

## 3. NLRP3 in porcine DNA virus infections

### 3.1. African swine fever virus (ASFV)

ASFV, a large enveloped dsDNA virus (family Asfarviridae, genome ~170–193 kb), encodes > 150 proteins and replicates predominantly in monocytes/macrophages, causing acute hemorrhagic disease with near-100% mortality in domestic pigs [66,67]. Its effects on NLRP3 run in both directions: separate viral proteins drive ion flux and priming, while others restrain assembly and transcription.

ASFV B169L is a viroporin localized to intracellular membranes, particularly the endoplasmic reticulum (ER), where it forms Ca^2+^-permeable channels. The disruption of Ca^2+^ homeostasis and induction of K^+^ efflux liberate NEK7, permitting NLRP3 assembly [68]. As depicted in Figure 2, mutagenesis identified residues 29, 55, and 57 within the transmembrane domain as critical for channel activity. Furthermore, silencing B169L attenuates both inflammasome activation and viral replication, indicating that ASFV exploits ion flux to support its replicative niche. The virus also enhances the priming phase. The viral protein C84L promotes NF-κB signaling by facilitating p65 phosphorylation, increasing transcription of NLRP3, pro-IL-1β, IL-6, and TNF-α and inducing pyroptosis Concurrently, pEP364R engages host DDX3X to potentiate caspase-1 activation and IL-1β maturation [33]. Although ASFV encodes multiple NF-κB antagonists, these pro-inflammatory activities likely operate in temporally and cell-type-dependent manner, contributing to the excessive inflammation characteristic of acute ASF.

**Figure 2.**
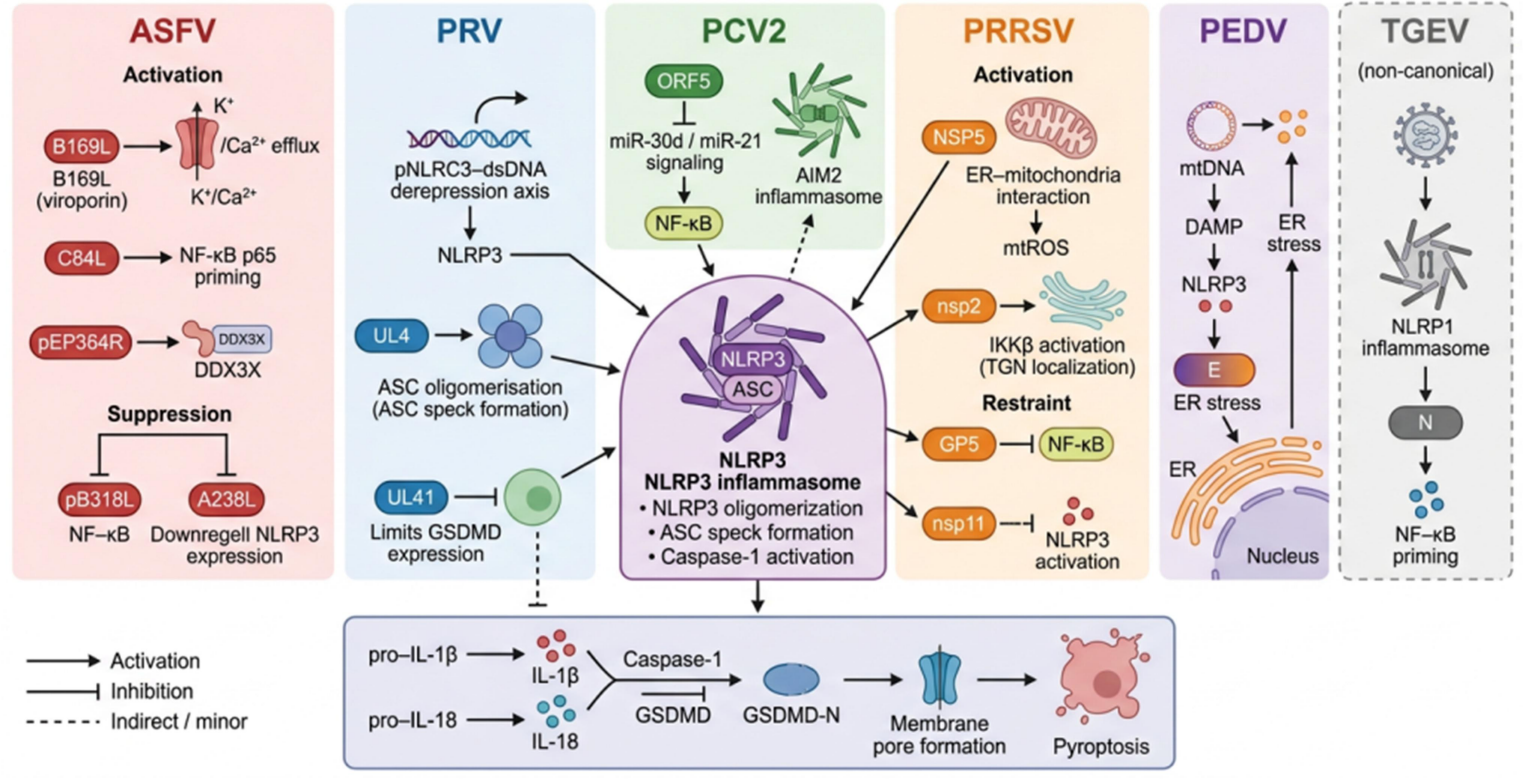
Modulation of the NLRP3 inflammasome by major swine viral pathogens. NLRP3 integrates activating and inhibitory signals from six major porcine viruses. ASFV activates NLRP3 via B169L (ion flux), C84L (NF-κB priming), and pEP364R (DDX3X), while pB318L and A238L inhibit this activation. PRV promotes activation through NLRC3–dsDNA derepression and UL4-induced ASC oligomerization. PCV2 primarily activates AIM2 and enhances NF-κB signaling via the ORF5–miR-30d–miR-21 axis. PRRSV activates NLRP3 via NSP5 (ER–mitochondria) and nsp2 (TGN), while GP5-induced mitophagy limits excessive activation. PEDV induces the mtDNA–DAMP axis and ER stress. TGEV (dashed) preferentially activates NLRP1. Downstream outputs include IL-1β/IL-18 secretion and pyroptosis.

ASFV further modulates NLRP3 activity by interfering with autophagy. The viral Bcl-2 homolog A179L binds Beclin-1, disrupting the VPS34–Beclin-1–ATG14 complex and inhibiting autophagosome initiation [69–71]. Mechanistically, impaired mitophagy sustains NLRP3-activating signals by preventing the clearance of damaged mitochondria, thereby prolonging activation duration and amplifying signaling intensity. This sustained presence of mtROS and mtDNA elevates cytokine release over time. Additionally, MGF360-4 L redirects the autophagy receptor p62/SQSTM1 toward MDA5 degradation, weakening antiviral sensing [72,73]. The viral protein A238L suppresses NF-κB- and NFAT-dependent transcription, reducing NLRP3 priming [74–77]. ASFV therefore activates some arms of the inflammasome pathway and restrains others, which allows it to set the level of macrophage inflammation (Figure 2).

### 3.2. Porcine circovirus type 2 (PCV2)

PCV2 is a small, non-enveloped ssDNA virus (~1.7 kb, family Circoviridae) that causes porcine circovirus-associated diseases (PCVAD), including post-weaning multisystemic wasting syndrome [78]. Despite its minimal genomic size, PCV2 reprograms host immune responses through several signaling nodes. Unlike many RNA viruses that rely heavily on ER stress, PCV2 induces profound mitochondrial dysfunction and endolysosomal damage following entry and uncoating. Cellular stress induced by PCV2 leads to the loss of mitochondrial membrane potential, generation of mtROS, and release of mtDNA into the cytosol [79]. These mitochondrial DAMPs directly engage the NLRP3 activation signal. Concurrently, cytosolic viral DNA intermediates are sensed by AIM2, leading to ASC oligomerization, caspase-1 activation, and IL-1β maturation. While the relative contributions of AIM2 vs NLRP3 to pathology remain partially unresolved, the convergence of mtDNA and viral DNA sensing ensures robust inflammasome assembly during PCV2 infection. PCV2 also acts through host microRNA networks. The viral ORF5 protein suppresses ssc-miR-30d, a nuclear microRNA that normally inhibits maturation of miR-21. Loss of miR-30d leads to sustained miR-21 upregulation, which enhances NF-κB activity and reinforces NLRP3 priming [80]. This indirect mechanism enables persistent inflammasome activation despite the virus’s minimal genome.

### 3.3. Pseudorabies virus (PRV)

PRV (Sus alphaherpesvirus 1) is an enveloped dsDNA virus (~143 kb) causing Aujeszky’s disease, characterized by acute encephalitis in non-swine hosts and latency in sensory ganglia [81]. It activates NLRP3 by linking viral DNA sensing to host regulatory pathways. Under basal conditions, porcine NLRC3 interacts with NLRP3 to suppress activation [82]. During infection, viral dsDNA binds NLRC3, disrupting the inhibitory complex and releasing NLRP3 for oligomerization. This is accompanied by increased ASC speck formation, caspase-1 activation, GSDMD cleavage, and IL-1β secretion. In parallel, the PRV UL4 protein directly enhances ASC oligomerization and speck formation, promoting pyroptosis [34]. This activity likely contributes to local inflammation and neuropathology. PRV also activates TLR–NF-κB signaling and the AIM2 inflammasome, indicating the concurrent operation of multiple inflammasome pathways [83].

## 4. NLRP3 in porcine RNA virus infections

RNA viruses predominantly engage NLRP3 through indirect mechanisms that converge on cellular stress pathways, including ER stress, mitochondrial dysfunction, ionic imbalance, and mtDNA release, rather than through direct ligand recognition [84].

### 4.1. Porcine reproductive and respiratory syndrome virus (PRRSV)

PRRSV, an enveloped positive-sense RNA arterivirus (~15 kb), targets porcine alveolar macrophages and causes reproductive failure, respiratory disease, and immunosuppression [85]. Its persistence is facilitated by extensive immune evasion, including suppression of interferon signaling and modulation of autophagy and ubiquitination [86–90]. Two nonstructural proteins push NLRP3 output up, while the envelope glycoprotein GP5 holds it below the lytic threshold.

PRRSV activates NLRP3 in macrophages via nonstructural protein NSP5, which recruits NLRP3 to ER–mitochondria contact sites. This relocation induces ER stress and Ca^2+^ leakage, providing the activation signal for inflammasome assembly [90]. The G30A mutation abolishes this activity and reduces IL-1β release. Simultaneously, NSP5 retains STING at the ER, preventing Golgi translocation and suppressing type I interferon production, thereby establishing a “high-inflammation, low-interferon” state conducive to viral persistence [72] (Figure 3). Priming is reinforced by a second nonstructural protein: PRRSV-2 nsp2 promotes NLRP3 priming through IKKβ-dependent dispersal of the trans-Golgi network (TGN) [35]. TGN disruption generates lipid-derived signals such as DAG, amplifying NF-κB-driven transcription and facilitating ASC oligomerization.

**Figure 3.**
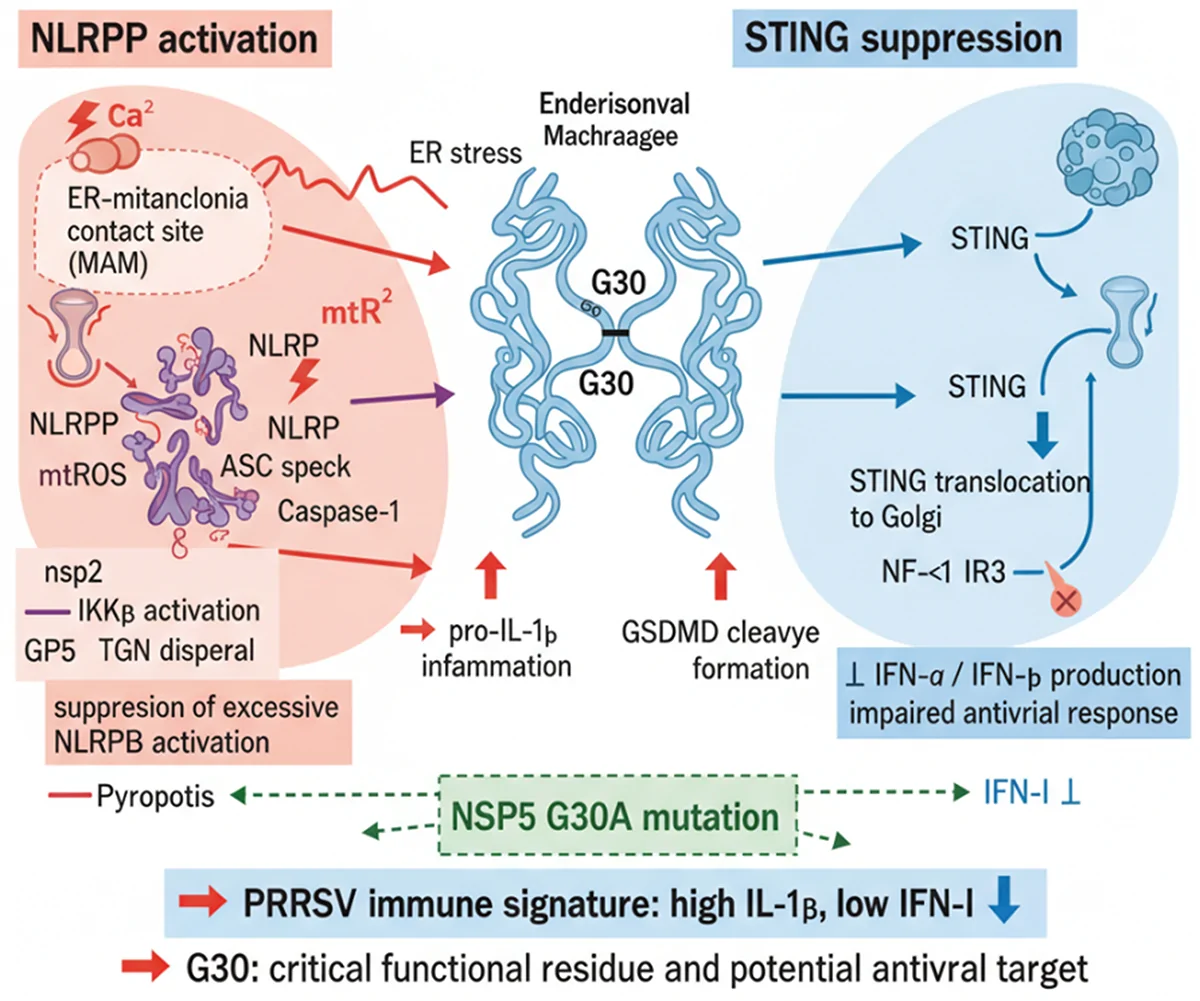
PRRSV NSP5 as a dual immune regulator: simultaneous NLRP3 activation and sting suppression.

In porcine alveolar macrophages, PRRSV NSP5 (ER-localized, key residue G30) exerts dual functions. Left panel: NSP5 recruits NLRP3 to ER–mitochondria contact sites, inducing ER stress, Ca^2+^release, and mtROS, leading to NLRP3 activation, IL-1β secretion, and pyroptosis. Right panel: NSP5 retains STING at the ER, blocking its translocation and suppressing type I interferon production. The G30A mutation disrupts both activities (dashed green box), identifying this residue as a potential antiviral target.

Countering these activating inputs, the envelope glycoprotein GP5 associates with mitochondria, increasing ROS production while simultaneously inducing mitophagy [91]. The clearance of damaged mitochondria reduces ROS-dependent signaling, preventing excessive NLRP3 activation and pyroptosis. This buffering mechanism enables sustained, sublethal inflammasome activation that supports viral dissemination without eliminating host cells. A further layer of control comes from ubiquitin-dependent interferon suppression: PRRSV NSP11 targets STAT2 for K48-linked ubiquitination and degradation, impairing interferon-stimulated gene expression [92]. Loss of interferon-induced negative regulators indirectly promotes NLRP3 activation by lowering the activation threshold, which links ubiquitin-mediated interferon suppression and enhanced inflammasome responsiveness.

### 4.2. Porcine epidemic diarrhea virus (PEDV)

PEDV, an enteric alphacoronavirus (~28 kb), infects intestinal epithelial cells and causes severe diarrhea with high mortality in neonatal piglets [93]. Infection induces mitochondrial dysfunction characterized by loss of membrane potential and accumulation of damaged mitochondria [38]. Here, mtROS release activates NF-κB for priming, while cytosolic mtDNA directly triggers NLRP3 assembly, leading to caspase-1 activation and IL-1β maturation. This mtDNA-driven pathway is central to intestinal inflammation and villous atrophy. The viral E protein, acting as a viroporin, likely contributes to this process by inducing ER stress and upstream mitochondrial damage [94]. ER stress adds a second activating input: PEDV activates the unfolded protein response via PERK and IRE1α pathways, and ER stress promotes NLRP3 activation through Ca^2+^ release, mitochondrial ROS amplification, and K^+^ efflux [37,38,95]. The convergence of ER stress and mtDNA signaling explains the robust IL-1β response despite viral suppression of interferon pathways.

PEDV encodes multiple antagonists of upstream signaling. The N protein inhibits TBK1–IRF3 activation, nsp1 disrupts the IRF3–CBP complex, and nsp2 induces degradation of FBXW7, destabilizing RIG-I and TBK1 [96–98]. These effects collectively dampen NF-κB-mediated priming while allowing DAMP-driven NLRP3 activation to proceed.

### 4.3. Transmissible gastroenteritis virus (TGEV)

TGEV is an alphacoronavirus (~28.6 kb) that targets intestinal epithelium, causing severe enteritis in neonatal piglets. Entry is mediated by S protein binding to porcine aminopeptidase N [99]. In contrast to other porcine RNA viruses, TGEV predominantly activates the NLRP1 inflammasome. Infection induces NLRP1-dependent caspase-1 activation and IL-1β secretion; inhibition of NLRP1, but not of NLRP3, significantly [100]. This highlights pathogen-specific inflammasome usage even among related coronaviruses. Inflammasome sensor usage is therefore pathogen-specific even among related coronaviruses. Priming still proceeds through NF-κB: the nucleocapsid protein activates NF-κB signaling, enhancing transcription of pro-inflammatory cytokines including IL-1β, IL-6, IL-8, TNF-α, and TGF-β, with the functional domain (aa 128–252) mediating this activity [101,102].

## 5. Viral antagonism of NLRP3 and immune evasion

Several viral proteins directly suppress NLRP3 assembly. ASFV pB318L inhibits both NF-κB activation and inflammasome formation [32], while A238L suppresses transcriptional priming via NF-κB and NFAT inhibition [75,103]. In PRRSV, NSP5 and nsp1α target multiple steps in NF-κB signaling, raising the activation threshold for NLRP3 [104] (Figure 2). Upstream signaling is attacked as well. Disruption of NF-κB and MAPK pathways limits NLRP3 priming. ASFV MGF505-7 R promotes TBK1 degradation and blocks p65 nuclear translocation [105]; PRRSV nsp1α inhibits IκBα phosphorylation [85]; and PEDV nonstructural proteins (nsp3, nsp5, nsp7, nsp14) suppress p65 activation through distinct mechanisms [97]. Transcriptional control is thus a recurrent node of immune evasion (Figure 2).

Some viruses interfere with pyroptosis downstream of inflammasome activation. PRV UL41-mediated host mRNA degradation may limit IFN expression [106]. In contrast, PEDV and TGEV readily induce pyroptosis in epithelial cells, indicating limited inhibition of GSDMD-mediated pore formation despite strong upstream suppression (Figure 2). PRRSV NSP5 shows how these activities can be combined in a single protein (Figure 3). By promoting ER stress-dependent NLRP3 activation while retaining STING at the ER to block interferon induction, NSP5 uncouples inflammatory and antiviral responses, establishing a permissive immune environment and identifying NSP5 as a potential antiviral target [104].

## 6. Porcine vs human NLRP3 inflammasomes: conservation, divergence, and model value

Pigs are used increasingly as translational models for human inflammatory and infectious disease, so the similarities and differences between the porcine and human NLRP3 systems matter for interpretation. The core inflammasome machinery, including NLRP3, ASC, and caspase-1, exhibits high sequence and functional conservation between pigs and humans [107]. Both species utilize the two-signal activation model (priming and activation) and rely on NEK7 for NLRP3 oligomerization [42,82,108]. Downstream execution via GSDMD cleavage to form pyroptotic pores is also functionally analogous, driving IL-1β/IL-18 release and lytic cell death [104,109].

Divergence appears mainly in the non-canonical pathway and in cytokine output. Humans possess caspase-4 and caspase-5, whereas pigs and rodents primarily rely on caspase-11 homologs for cytosolic LPS sensing [53]. The regulatory nuances of porcine non-canonical caspases in viral infections, especially regarding their threshold for activation by endotoxin or viral-induced membrane damage, remain poorly defined and warrant caution when extrapolating human data to swine models [110]. Transcriptional regulation and cytokine profiles also differ in ways that can influence disease outcome. For instance, porcine macrophages often exhibit distinct interferon kinetics and IL-1β secretion magnitudes following viral stimulation compared to human monocyte-derived macrophages [38,107]. Furthermore, while pyroptosis is conserved, the relative contribution of NLRP3-induced immunopathology vs protective antiviral immunity may differ, partly due to variations in host restriction factors and receptor distributions [111] (e.g. CD163 in PRRSV infection).

Pigs are highly suitable models for human inflammasome-associated diseases due to their physiological, anatomical, and immunological similarities to humans, far surpassing murine models in certain contexts [112–114] (e.g. respiratory and enteric physiology). However, limitations exist: swine-specific viral evasion proteins (e.g. ASFV A238L, PRRSV NSP5) target porcine-specific immune pathways, which may not perfectly recapitulate human disease pathogenesis [75,115,116]. Mechanistic insight from swine viruses is therefore informative about inflammasome regulation but needs careful contextualization before it is applied to human medicine. Within those limits, porcine data speak directly to human questions, particularly in understanding how inflammasome modulation affects tissue-specific immunopathology in respiratory and gastrointestinal tracts [117]. Conversely, human-specific NLRP3 pharmacology (e.g. MCC950) can be effectively tested in swine models to evaluate therapeutic windows before clinical translation [118,119]. A summary of major comparative findings is provided in Supplementary Table 2.

## 7. The ubiquitin–autophagy–NLRP3 axis as an integrating hub

Crosstalk among NLRP3, autophagy, and ubiquitination forms a central regulatory axis shaping inflammasome activity during swine viral infections (Figure 4). Selective autophagy, mediated by cargo receptors such as p62/SQSTM1, NDP52, and NBR1, links K63-ubiquitinated substrates to autophagosomes, limiting inflammasome activation through removal of damaged mitochondria and degradation of inflammasome-inducing viral components [120,121]. In parallel, the ubiquitin–proteasome system exerts direct control over NLRP3 stability: K48-linked ubiquitination promotes proteasomal degradation, whereas K63-linked ubiquitination enhances inflammasome assembly competence [59,60].

**Figure 4.**
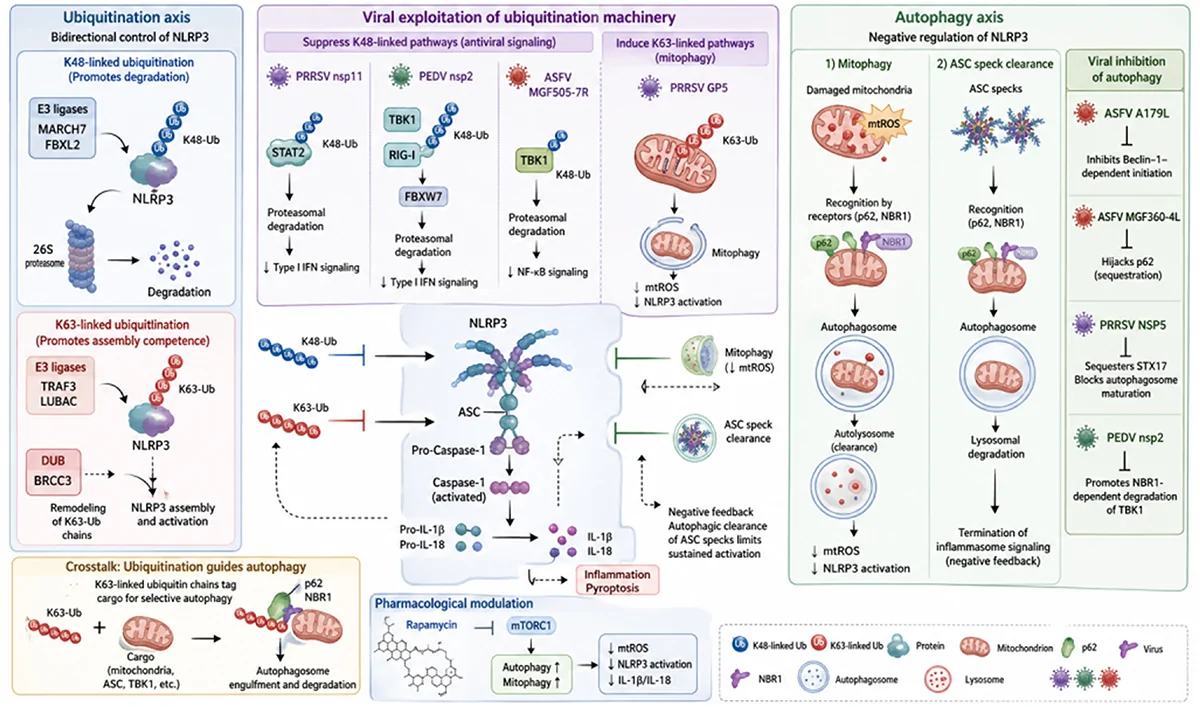
Interplay between the NLRP3 inflammasome, ubiquitination, and autophagy during swine viral infections.

Three regulatory modules control NLRP3 activity. Ubiquitination axis (left): K48-linked ubiquitination targets NLRP3 for proteasomal degradation; K63-linked ubiquitination promotes assembly. Viral exploitation of this axis includes PRRSV nsp11 (STAT2 degradation), PEDV nsp2 (FBXW7 degradation), and ASFV MGF505-7 R (TBK1 degradation). Autophagy axis (right): Mitophagy clears damaged mitochondria; ASC speck autophagy provides negative feedback. Viral interference include ASFV A179L (Beclin-1 inhibition), PRRSV NSP5 (STX17 sequestration), and PEDV nsp2 (NBR1-mediated TBK1 degradation). Rapamycin is indicated as a candidate modulator of autophagic flux.

Porcine viruses exploit this network with notable specificity. ASFV MGF360-4 L redirects p62/SQSTM1 toward MDA5 degradation [72]. PRRSV NSP5 disrupts autophagosome–lysosome fusion via sequestration of syntaxin-17, leading to accumulation of immature autophagosomes that serve as replication niches while sustaining mitochondrial stress and low-level NLRP3 activation [122]. PEDV nsp2 reprograms selective autophagy through NBR1-dependent degradation of TBK1 [98]. These observations support a model wherein NLRP3 functions as a convergence node integrating mitochondrial damage, metabolic stress, and disrupted proteostasis. Dissecting causality within this triad will require genetic knockout swine models and temporal perturbation approaches.

## 8. Therapeutic implications, knowledge gaps, and future directions

### 8.1. Pharmacological targeting of NLRP3 and pyroptosis execution

The dual role of NLRP3 in host protection and immunopathology presents both opportunities and challenges. MCC950 (CP-456773), which stabilizes the inactive NACHT conformation of NLRP3, shows robust anti-inflammatory efficacy in multiple disease models [123]. Extrapolation to swine viral infections suggests potential utility in limiting tissue damage during PRRSV-associated pneumonia, PEDV/TGEV-induced enteritis, and acute ASF. However, broad inhibition may compromise antiviral immunity, as NLRP3-mediated pyroptosis eliminates infected cells and IL-18 activates NK and CD8^+^ T cells. Identifying the optimal therapeutic window in swine infection models remains a priority.

An alternative is to uncouple cytokine maturation from membrane rupture by targeting GSDMD represents an alternative strategy. Compounds such as disulfiram and necrosulfonamide inhibit pore formation by modifying GSDMD-N, limiting tissue damage without fully suppressing upstream inflammasome signaling [124]. Virus-specific differences in pyroptotic responses (e.g. pronounced pyroptosis in PEDV/TGEV/PRV vs sublytic activation in PRRSV) complicate this approach and necessitate *in vivo* validation.

### 8.2. Virus- and host-directed targets: the NSP5–STING axis and proteostasis

PRRSV NSP5 is a compelling target due to its bifunctional role. Selective disruption of NSP5–STING interaction could restore interferon responses without exacerbating inflammasome-driven pathology. Conversely, blocking NSP5-mediated recruitment of NLRP3 to the ER–mitochondria interface may reduce inflammatory damage. Structure-guided drug design targeting the NSP5–STING interface is worth pursuing. Host-directed interventions targeting autophagy or ubiquitination may offer nuanced control. mTOR inhibitors (e.g. rapamycin) enhance autophagic flux and suppress NLRP3 activation via mitophagy, though pleiotropic effects necessitate careful evaluation in swine systems [125]. Targeting deubiquitinases that regulate K63-linked signaling could modulate inflammasome activation, but current lack of specificity among DUB inhibitors presents translational challenges.

### 8.3. Knowledge gaps and research priorities

Despite recent advances, several critical gaps remain in our understanding of NLRP3 regulation in swine viral infections. First, most current data derive from non-porcine or immortalized cell systems; porcine NLRP3 may exhibit distinct regulatory features that require swine-specific validation using primary cells [33,38,107,126]. Second, while the dominance of NLRP1 in TGEV infection is established, the potential involvement of non-canonical inflammasome pathways and specific sensor usage in related enteric coronaviruses like PEDV remains largely unexplored [38,100]. Third, the delicate balance between protective and pathological inflammasome responses needs precise definition through controlled in vivo infection models [127–129]. Fourth, systematic identification of novel viral antagonists, particularly in ASFV, is necessary to fully map the host-pathogen interface [32,130]. Finally, the adjuvant potential of controlled NLRP3 activation in antigen-presenting cells offers a promising avenue for improving swine vaccine efficacy, an area that remains empirically underexplored [131–133].

## 9. Conclusion

The NLRP3 inflammasome plays a paradoxical role in host responses to major swine viral pathogens. Across ASFV, PRRSV, PRV, PEDV, TGEV, and PCV2, viruses engage this pathway through distinct upstream triggers while simultaneously deploying countermeasures to prevent excessive pyroptotic loss of their replication niche. Viral strategies range from ASFV viroporins and NF-κB modulators to PRRSV NSP5-driven ER–mitochondria signaling, which shows how flexible inflammasome regulation can be. Convergence on the ubiquitin–autophagy–NLRP3 network makes this axis both a liability for the host and a point of intervention. Translational exploitation will depend on integrating mechanistic studies in physiologically relevant systems with in vivo infection models and veterinary-specific pharmacology.

## Supplementary Material

Clean Copy of Suppelementary Tables - QVIR-2026-0492.R2.docx

## Data Availability

There is no data associated with this research.

## Notes

This study affirms that neither generative artificial intelligence nor any other AI-assisted technologies were employed in the preparation of this manuscript.
