# Supplementary material for: NLRP3 inflammasome in major swine viral infections: Host defense and viral antagonism
Source: Virulence. 2026 Oct 29;17(1):2753323. doi: 10.1080/21505594.2026.2753323 (PMC13733211; doi:10.1080/21505594.2026.2753323)
Supplement: Supplementary file 1 [file KVIR_A_2753323_SM0781.docx]

**Supplementary Table 1. Summary of viral proteins targeting the NLRP3 inflammasome in major swine pathogen**

| **Virus** | **Viral Protein** | **Mechanism** | **Effect on NLRP3** | **References** |
| --- | --- | --- | --- | --- |
| ASFV  ASFV  ASFV  ASFV  ASFV  PRV  PRV  PRV  PCV2  PRRSV  PRRSV  PRRSV  PRRSV | B169L  pEP364R  A179L  pB318L  A238L  dsDNA  UL4  UL41  ORF5  NSP5  NSP2  GP5  NSP11 | Ca²⁺-permeable viroporin, K⁺ efflux  DDX3X interaction  Beclin-1 binding, autophagy inhibition  NF-κB and inflammasome inhibition  NF-κB/NFAT inhibition  NLRC3 binding, derepression  ASC oligomerization  IFN-β expression limitation  miR-30d suppression → miR-21↑ → NF-κB↑  ER-mitochondria recruitment  IKKβ-dependent TGN dispersal  Mitophagy induction  STAT2 K48 ubiquitination | Activation  Activation  Sustains activation  Suppression  Suppression  Activation  Activation  Suppression  Priming  Activation  Priming  Suppression  Indirect promotion via lowering the activation threshold | ^59^  ^26^  ^60^  ^25^  ^67, 68^  ^72^  ^27^  ^103^  ^70^  ^94^  ^28^  ^81^  ^82^ |

**Supplementary** **Table 2. Summary of major comparative findings in pigs versus humans.**

| **Feature** | **Pigs (Swine)** | **Humans** | **Translational Implication** |
| --- | --- | --- | --- |
| Core Machinery | High conservation of NLRP3, ASC, Caspase-1, NEK7 | High conservation of NLRP3, ASC, Caspase-1, NEK7 | Pigs serve as robust models for studying fundamental NLRP3 assembly and pyroptosis |
| Canonical Activation | Two-signal model (Priming via NF-κB; Activation via K⁺ efflux, mtROS, mtDNA) | Two-signal model identical in pathway architecture | Therapeutics targeting canonical activation (e.g., MCC950) can be effectively preclinically tested in swine. |
| Non-Canonical Pathway | Relies on caspase-11 homologs for cytosolic LPS sensing | Utilizes caspase-4 and caspase-5. | Differences in non-canonical caspase homologs require caution when extrapolating endotoxin-mediated shock models between species. |
| Viral Evasion Targets | Swine-specific viral proteins (e.g., PRRSV NSP5, ASFV A238L) target porcine macrophage pathways | Human-specific viral evasion (e.g., SARS-CoV-2 proteins) targets human macrophage pathways. | Mechanistic insights are highly valuable, but species-specific viral evasion strategies limit direct 1:1 pathological comparison. |
| Physiology & Immunopathology | Respiratory and enteric anatomy highly similar to humans; robust macrophage responses. | Standard clinical reference. | Swine models are superior to murine models for studying tissue-specific immunopathology in respiratory (PRRSV) and enteric (PEDV/TGEV) viral diseases. |
